# Safety and feasibility of minimally invasive gastrectomy following preoperative chemotherapy for highly advanced gastric cancer

**DOI:** 10.1186/s12876-024-03155-5

**Published:** 2024-02-15

**Authors:** Tsuyoshi Tanaka, Koichi Suda, Susumu Shibasaki, Akiko Serizawa, Shingo Akimoto, Masaya Nakauchi, Hiroshi Matsuoka, Kazuki Inaba, Ichiro Uyama

**Affiliations:** 1 Department of Surgery, Fujita Health University, 1-98 Dengakugakubo, Kutsukake, Toyoake, Aichi 470-1192 Japan; 2 Collaborative Laboratory for Research and Development in Advanced Surgical Intelligence, Fujita Health University, Toyoake, Japan; 3 Department of Advanced Robotic and Endoscopic Surgery, Fujita Health University, Toyoake, Japan; 4 Collaborative Laboratory for Research and Development in Advanced Surgical Technology, Fujita Health University, Toyoake, Japan

**Keywords:** Stomach neoplasms, Neoadjuvant chemotherapy, Induction chemotherapy, Minimally invasive surgery

## Abstract

**Background:**

This study aimed to determine the safety and feasibility of minimally invasive gastrectomy in patients who underwent preoperative chemotherapy for highly advanced gastric cancer.

**Methods:**

Preoperative chemotherapy was indicated for patients with advanced large tumors (≥ cT3 and ≥ 5 cm) and/or bulky node metastasis (≥ 3 cm × 1 or ≥ 1.5 cm × 2). Between January 2009 and March 2022, 150 patients underwent preoperative chemotherapy followed by gastrectomy with R0 resection, including conversion surgery (robotic, 62; laparoscopic, 88). The outcomes of these patients were retrospectively examined.

**Results:**

Among them, 41 and 47 patients had stage IV disease and underwent splenectomy, respectively. Regarding operative outcomes, operative time was 475 min, blood loss was 72 g, morbidity (grade ≥ 3a) rate was 12%, local complication rate was 10.7%, and postoperative hospital stay was 14 days (Interquartile range: 11–18 days). Fifty patients (33.3%) achieved grade ≥ 2 histological responses. Regarding resection types, total/proximal gastrectomy plus splenectomy (29.8%) was associated with significantly higher morbidity than other types (distal gastrectomy, 3.2%; total/proximal gastrectomy, 4.9%; *P* < 0.001). Specifically, among splenectomy cases, the rate of postoperative complications associated with the laparoscopic approach was significantly higher than that associated with the robotic approach (40.0% vs. 0%, *P* = 0.009). In the multivariate analysis, splenectomy was an independent risk factor for postoperative complications [odds ratio, 8.574; 95% confidence interval (CI), 2.584–28.443; *P* < 0.001].

**Conclusions:**

Minimally invasive gastrectomy following preoperative chemotherapy was feasible and safe for patients with highly advanced gastric cancer. Robotic gastrectomy may improve surgical safety, particularly in the case of total/proximal gastrectomy combined with splenectomy.

**Supplementary Information:**

The online version contains supplementary material available at 10.1186/s12876-024-03155-5.

## Introduction

Gastric cancer is the fourth leading cause of death worldwide [1]. Radical gastrectomy plays a major role in the curative treatment of gastric cancer [2]; however, the prognosis of patients with highly advanced gastric cancer remains unsatisfactory.

To improve the prognosis, perioperative chemotherapy or chemoradiotherapy has been applied to patients with advanced gastric cancer, particularly in Western countries [3–5]. The efficacy of preoperative chemotherapy followed by gastrectomy has recently been reported in clinical trials [6–8]. In Japan, preoperative chemotherapy for highly advanced gastric cancer with para-aortic lymph node metastasis has been associated with favorable outcomes [9]. Nonetheless, preoperative chemotherapy has not been established as the standard treatment due to the better outcomes obtained after D2 gastrectomy plus adjuvant chemotherapy than those reported in Western countries [10–12]. Thus, the use of neoadjuvant chemotherapy for resectable gastric cancer remains under debate in Japan.

Technological advances have allowed the use of minimally invasive surgery for early- and advanced-stage gastric cancer. This approach has shown non-inferiority to open surgery in clinical trials [13–15]. However, open surgery is still the standard treatment in Japan, for the cases after preoperative chemotherapy as previously reported [9–12]. Thus far, we have demonstrated the feasibility and safety of robotic gastrectomy (RG) compared with laparoscopic gastrectomy (LG) [16–19]. Previous studies of RG documented favorable short-term outcomes; however, the majority of patients included in these investigations had clinical cancer stage (cStage) 1–2 disease [20–22]. To date, there exists little data regarding minimally invasive surgery, including RG, for patients with highly advanced gastric cancer who underwent preoperative chemotherapy [23–25]. Notably, in the surgery after preoperative chemotherapy (including conversion surgery from cStage IV), fibrosis, and disruption of the anatomical structure may complicate dissection, resulting in more blood loss during operation and longer operative time [26].

Therefore, in this study, we aimed to confirm the safety and feasibility of minimally invasive gastrectomy for patients with highly advanced gastric cancer who underwent preoperative chemotherapy, and evaluate the clinical advantage of RG over LG.

## Materials and methods

### Patients

The retrospective study was conducted in a single institution using a prospectively maintained database between January 2009 and March 2022. At Fujita Health University Hospital (Toyoake, Aichi, Japan), 261 consecutive gastric cancer patients, including cStage IV underwent neoadjuvant or induction chemotherapy. Of those, 150 patients who met the following indications of preoperative chemotherapy and successful R0 resection minimal invasively were selected for data analysis (Supplemental Fig. 1). The indications of chemotherapy were as follows: 1) histologically confirmed primary gastric adenocarcinoma; 2) detection of an advanced large tumor (≥ cT3 and ≥ 5 cm) or bulky node metastasis (≥ 3 cm × 1 or ≥ 1.5 cm × 2). We defined these indications plus cStageIV as highly advanced gastric cancer because of their poor prognosis [9, 27]. In this study, when R0 resection was achieved, cStage IV patients with R0 resection were analyzed together with cStage II–III patients due to favorable outcomes [25].

Routine preoperative evaluation to determine operability included a complete blood count, serum chemistry, electrocardiography, spirometry, oxygen saturation (SpO_2_), activated partial thromboplastin time, and prothrombin time. Patients who met the following criteria were considered eligible for preoperative chemotherapy followed by radical gastrectomy under general anesthesia: Eastern Cooperative Oncology Group performance status (PS) ≤ 1; adequate hematologic (white blood cell count ≥ 4000/mm^3^ and ≤ 12,000/mm^3^; neutrophil count ≥ 2000/mm^3^; hemoglobin levels ≥ 8.0 g/dL; platelet count ≥ 100,000/mm^3^), cardiac (ejection fraction ≥ 50%), respiratory function (SpO_2_ ≥ 95% and forced expiratory volume in 1 s > 1.5 L), hepatic status (aspartate aminotransferase and alanine aminotransferase levels ≤ 100 IU/L; total bilirubin levels ≤ 1.5 mg/dL), and renal function (creatinine levels ≥ 1.2 mg/dL and creatinine clearance rate ≥ 60 mL/min).

Patients were involved in all treatment decisions and provided informed consent. This study was approved by the Institutional Review Board of Fujita Health University (HM20-265).

### Treatment decision-making process

Tumor staging was evaluated by endoscopy, upper gastrointestinal series, computed tomography, abdominal ultrasonography, and positron emission tomography (if necessary), as per the 15th edition of the Japanese Classification of Gastric Carcinoma [28]. Treatment decision was discussed by experienced upper gastroenterological surgeons and physicians on a weekly basis. The patients who had been selected to receive preoperative chemotherapy underwent staging laparoscopy before the initiation of chemotherapy for the detection of potential peritoneal seeding. All patients received preoperative chemotherapy as follows. The standard therapeutic regimen administered in these patients was S-1 (i.e., tegafur, gimeracil, and oteracil potassium) plus cisplatin (SP) [29], or S-1 plus oxaliplatin (SOX) [30], alternatively, docetaxel/cisplatin/S-1 (DCS) [31] or capecitabine/cisplatin (XP) [32] (or SOX) plus trastuzumab, XP (SOX) + trastuzumab [33]; regimens were adopted as clinical practice following an expert consensus based on the tumor characteristics. Planned surgery for resectable cStage II–III gastric cancer was scheduled after two cycles of SP or three cycles of SOX. Conversion surgery from stage IV gastric cancer was performed only after confirmation of noncurable factors by preoperative examination, such as positron emission tomography-computed tomography and/or repeated staging laparoscopy.

The surgical approach for RG or LG was decided as follows. During the study period, RG was not covered by national medical insurance (until March 2018). Hence, 16 patients who had agreed to undergo RG using the da Vinci Surgical System (Intuitive Surgical Inc, USA) had to pay 2,200,000 JPY upon perioperative admission to undergo RG, whereas 84 patients who had not agreed to be treated with RG underwent LG covered by medical insurance. Since April 2018, RG covered by medical insurance has been the primary choice for gastrectomy at our institute.

### Surgery

The surgical procedure was performed based on the concept of outermost layer-oriented nodal dissection, as previously reported [34]. Regarding the energy device, laparoscopic coagulating shears were mainly adopted in LG, whereas the Maryland bipolar forceps (Intuitive Surgical Inc, USA) using the Macro bipolar mode at 60 W (Force Triad™ energy platform; Medtronic, Minneapolis, MN, USA) was mainly employed in RG [35]. Splenectomy was performed in cases with tumor invasion of the greater curvature or presence of a clinically positive No. 10 lymph node. Since January 2019, we utilized near-infrared fluorescence imaging with indocyanine green for splenectomy using the Firefly of da Vinci Xi system (Intuitive Surgical Inc, USA), which enabled us to confirm the blood perfusion of the pancreatic tail to avoid the occurrence of a refractory pancreatic fistula caused by ischemia [36]. The surgeon’s qualification for laparoscopic gastrectomy was as follows; 1) qualified by the Japanese Society of Endoscopic Surgery endoscopic surgical skill qualification system; and 2) experience with > 50 LGs. For RG, the surgeon’s qualification was as follows: 1) certified as a da Vinci Surgical System (DVSS) console surgeon; 2) certified by the Japanese Society of Gastroenterological Surgery; 3) qualified by the Japanese Society of Endoscopic Surgery endoscopic surgical skill qualification system; and 4) approval of the proctor qualification by the Japanese Society of Endoscopic Surgery (i.e., > 40 RGs). The entire surgery was performed or guided by highly experienced surgeons (either I.U. or K.S.) (i.e., > 100 totally laparoscopic D2 gastrectomy procedures).

### Outcomes of interest

Clinicopathological features, postoperative short-term results, and long-term outcomes were examined. The risk factors of postoperative complications were also evaluated. Postoperative complications were graded according to the Clavien–Dindo (CD) classification [37, 38]. Any grade ≥ IIIa complication was considered clinically significant. Mortality was defined as CD grade V within 30 days after gastrectomy. Patients who exhibited grade ≥ 2 histological response of the primary lesion were defined as good responders [grade 0 (no effect): no evidence of effect; grade 1a (very slight effect): viable tumor cells occupying > 2/3 of the tumorous area; grade 1b (slight effect): viable tumor cells remain in > 1/3, but < 2/3 of the tumorous area; grade 2 (considerable effect): viable tumor cells remain in < 1/3 of the tumorous area; grade 3 (complete response): no viable tumor cells remain] [28]. Overall survival (OS) time and relapse-free survival (RFS) time were evaluated to assess the long-term outcomes. OS time was defined as the time from the date of diagnosis of gastric cancer to death due to any reason or interruption of the follow-up. RFS was calculated as the time between the date of gastrectomy and that of death due to any reason or the time when recurrence was detected.

### Statistical analysis

Statistical analysis was performed using IBM SPSS Version 22.0 J for Windows (IBM Corp., Armonk, NY, USA). The chi-squared test, Fischer’s exact test, Mann–Whitney *U* test, Kruskal–Wallis test, and logistic regression analysis were used, as appropriate. Long-term outcomes were analyzed using the Kaplan–Meier method with the log-rank test and Cox proportional hazards regression model. Data are expressed as the median [interquartile range (IQR)] unless otherwise stated. *P*-values < 0.05 (two-tailed) indicate statistically significant differences.

## Results

### Patient characteristics

The clinicopathological features of all 150 eligible patients who received preoperative chemotherapy and underwent gastrectomy are summarized in Table 1. Among the 150 patients treated with minimally invasive surgery, 62 patients (41.3%) underwent RG. The resection types were as follows: distal gastrectomy (DG; *n* = 62); total/proximal gastrectomy (TG/PG; *n* = 41); and total/proximal gastrectomy plus splenectomy (TG/PG + S; *n* = 47 [RG: *n* = 12; LG: *n* = 35]). Conversion surgery from cStage IV was performed in 47 patients, and R0 resection was achieved in 41 patients (RG: *n* = 23; LG: *n* = 18), whereas R1 was observed in 6 patients (2 patients each; proximal margin + , resectable margin + , and CY1, respectively). The histological response is shown in Table 1. Grade ≥ 2 histological response was achieved in 50 patients (33.3%). Adjuvant chemotherapy was administered in 126 patients (84.0%).

**Table 1:** Clinicopathological features

	Total (*n* = 150)
Age (years)	66 (59–71)
Sex, male:female	107:43
Body mass index (kg/m^2^)	21.8 (19.8–24.1)
ASA-PS^a^, 1:2:3	65:76:9
Tumor diameter (< 5:5–8: ≥ 8) (cm)	8:78:64
cT, 1b:2:3:4a:4b	1:10:40:94:5
cN, 0:1	37:113
cStage (TNM), II:III:IV	29:80:41
Approach, laparoscopic:robotic [da Vinci S/Si/Xi]	88:62 [8/8/46]
Type of resection, DG^b^:(TG^c^/PG^d^):(TG/PG + splenectomy)	62:41:47
Extent of lymphadenectomy, D1 + :D2:D2 +	7:138:5
Esophagogastric junctional cancer, n (%)	22 (14.7)
Conversion surgery, n (%)	41 (27.3)
Chemotherapy line	1 (1–1)
Chemotherapy regimen	
*SP*^*e*^*/SOX*^*f*^*/FOLFOX*^*g*^*/FP*^*h*^	*114 [67/42/4/1]*
*DCS*^*i*^	*20*
*XP*^*j*^*/SOX* + *Trastuzumab*	*10 [5/5]*
*Others[FLOT*^*k*^ ± *Durvalumab/S1* + *Paclitaxel iv*^*l*^*,ip*^*m*^*]*	*6 [4/2]*
Chemotherapy cycle	2 (2–4)
ypStage (TNM), 0:I:II:III:IV	12:28:52:56:2
Histological response (primary lesion), grade 0:1a:1b:2:3	6:56:38:36:14
*Grade* ≥ *2, n(%)*	*50 (33.3)*
Adjuvant chemotherapy (yes), n(%)	126 (84.0)
*S-1/Capecitabine*	*71 [69/2]*
*SP/SOX/CapeOX*^*n*^*/FP/FOLFOX*	*32 [9/20/1/1/1]*
*DS*^*o*^*/S-1* + *Paclitaxel*	*7 [5/2]*
*Docetaxel/nabPaclitaxel/CPT-11*	*6 [3/1/2]*
*XP/SOX* + *Trastuzumab*	*3 [2/1]*
*DCS*	*1*
*CPT-11* + *CDDP*	*1*
*Others[FLOT* ± *Durvalumab/unknown]*	*5 [3/2]*

Data are shown as median with interquartile range

^a^ASA-PS-American Society of Anesthesiologists-Physical Status

^b^DG-Distal gastrectomy

^c^TG-Total gastrectomy

^d^PG-Proximal gastrectmy

^e^SP-S-1 + cisplatin

^f^SOX-S-1 + oxaliplatin

^g^FOLFOX- 5-fluorouracil + leucovorin + oxaliplatin

^h^FP-5-fluorouracil + cisplatin

^i^DCS-Docetaxel + cisplatin + S-1

^j^XP-Capecitrabine + cisplatin

^k^FLOT-5-fluorouracil + leucovorin + oxaliplatin + docetaxel

^l^iv-Intravenously

^m^ip-Intraperitoneally

^n^CapeOX-Capecitrabine + oxaliplatin

^o^DS-Docetaxel + S-1

### Preoperative chemotherapy

As preoperative chemotherapy, the combination regimen of 5-fluorouracil and platinum-based anticancer agents [SP, SOX, FOLFOX (5-fluorouracil, and leucovorin plus oxaliplatin), 5-fluorouracil plus cisplatin (FP)] was mainly used. On the other hand, the triplet regimen (i.e., DCS) and trastuzumab-included regimen (XP or SOX plus trastuzumab) were administered as a clinical practice. In addition, other regimens [FLOT (5-fluorouracil, leucovorin, oxaliplatin, docetaxel) ± durvalumab; S-1 plus paclitaxel intravenously/intraperitoneally] were administered to patients who were registered in other clinical trials.

Treatment-related adverse events are summarized in Supplementary Table 1. Regarding hematological toxicity, neutropenia (grade ≥ 3) was the most frequently recorded adverse event (21.3%). Regarding non-hematological toxicity, gastrointestinal symptoms, diarrhea, anorexia, and nausea were the most commonly observed adverse events.

### Operative outcomes and postoperative complications

Descriptive results for the enrolled patients are summarized in supplementary Table 2. Regarding operative outcomes, postoperative complications (grade ≥ 3a) were observed in 18 patients (12.0%). Among those, a local complication was observed in 16 patients (10.7%), and pancreatic fistula was the most common complication (11 patients; 7.3%). The median hospital stay after surgery was 14 days.

The operative results stratified by resection type and approach (RG/LG) are summarized in Table 2. Significant differences were observed in the approach, number of operators, operative time, blood loss, and number of dissected nodes among the three groups. Morbidity was significantly higher with TG/PG + S (29.8%) versus others (DG 3.2%, TG/PG 4.9%, *P* < 0.001). Remarkably, in patients who underwent splenectomy, the rate of postoperative complications was significantly higher for the laparoscopic approach versus the robotic approach (LG 40.0% vs. RG 0%, *P* = 0.009). However, there was no difference between the two groups in those who did not undergo splenectomy (LG 3.8% vs. RG 4.0%, *P* = 1.000).

**Table 2:** Operative results stratified by resection type and approach

**Resection type**	DG^a^ (*n* = 62)	TG/PG^b^ (*n* = 41)	TG/PG + S^c^ (*n* = 47)	*P*-value
Approach, robotic:laparoscopic	26:36	24:17	12:35	0.007
Number of surgeons	18	12	9	0.025
Number of cases operated by each surgeon	2.5 (1–17)*	2 (1–15)*	4 (1–12)*	0.347
Operative time (min)	359 (309–435)	560 (446–669)	541 (501–624)	< 0.001
Blood loss (g)	46 (19–103)	70 (30–170)	138 (66–338)	< 0.001
Number of dissected nodes	37 (29–45)	39 (32–52)	47 (38–60)	< 0.001
In-hospital mortality	0	0	0	-
Morbidity (grade ≥ 3a), n (%)	2 (3.2)	2 (4.9)	14 (29.8)	< 0.001
Systemic complications, n (%)	1(1.6)	2 (4.9)	2 (4.3)	0.612
*Pneumonia*	*1*	*1*	*2*	*-*
*Pulmonary thromboembolism*	*0*	*0*	*1*	*-*
*Sepsis*	*0*	*0*	*1*	*-*
*Renal failure*	*0*	*1*	*0*	*-*
Local complications, n (%)	2 (3.2)	1 (2.4)	13 (27.7)	< 0.001
*Pancreatic fistula*	*1*	*0*	*10*	*-*
*Ileus*	*1*	*1*	*1*	*-*
*Anastomotic leakage*	*0*	*0*	*2*	*-*
*Abdominal abscess*	*1*	*0*	*1*	*-*
*Choledochiarctia*	*1*	*0*	*0*	*-*
*Diaphragmatic hernia*	*0*	*0*	*1*	*-*
Local infectious complications, n (%)	1(1.6)	0	11 (23.4)	< 0.001
Hospital stay following surgery (days)	12 (10–16)	13 (11–18)	19 (15–37)	< 0.001
**Nonsplenectomy cases**	Total (*n* = 103)	RG^d^ (*n* = 50)	LG^e^ (*n* = 53)	*P*-value
Number of surgeons	18	6	18	< 0.001
Number of cases operated by each surgeon	3 (1–32)*	5 (1–23)*	2 (1–9)*	0.137
Operative time (min)	421 (331–540)	458 (373–599)	361 (309–472)	< 0.001
Blood loss (g)	50 (20–112)	51 (26–113)	49 (19–119)	0.552
Number of dissected nodes	38 (29–46)	39 (29–48)	37 (30–46)	0.707
Morbidity (grade ≥ 3a), n (%)	4 (3.9)	2 (4.0)	2 (3.8)	1.000
Systemic complications, n (%)	3 (2.9)	2 (4.0)	1 (1.9)	0.610
Local complications, n (%)	3 (2.9)	2 (4.0)	1 (1.9)	0.610
* Pancreatic fistula*	*1*	*1*	*0*	*-*
* Ileus*	*2*	*1*	*1*	*-*
* Abdominal abscess*	*1*	*1*	*0*	*-*
* Choledochiarctia*	*1*	*1*	*0*	*-*
Local infectious complications, n (%)	1 (1.0)	1 (2.0)	0	-
Hospital stay following surgery (days)	13 (10–16)	13 (10–16)	13 (10–16)	0.811
**Splenectomy cases**	All (*n* = 47)	RG (*n* = 12)	LG (*n* = 35)	*P*-value
Number of operators	9	3	8	< 0.001
Number of cases operated by each surgeon	4 (1–12)*	3 (3–6)*	3 (1–10)*	0.921
Operative time (min)	541 (501–624)	604 (534–678)	523 (486–610)	0.064
Blood loss (g)	138 (66–338)	145 (70–246)	132 (53–413)	0.714
Number of dissected nodes	47 (38–68)	50 (40–72)	47 (36–64)	0.373
Morbidity (grade ≥ 3a), n (%)	14 (29.8)	0	14 (40.0)	0.009
Systemic complications, n (%)	2 (4.3)	0	2 (5.7)	1.000
Local complications, n (%)	13 (27.7)	0	13 (37.1)	0.021
* Pancreatic fistula*	*10*	*0*	*10*	*-*
* Ileus*	*1*	*0*	*1*	*-*
* Anastomotic leakage*	*2*	*0*	*2*	*-*
* Abdominal abscess*	*1*	*0*	*1*	*-*
* Diaphragmatic hernia*	*1*	*0*	*1*	*-*
Local infectious complications, n (%)	11 (23.4)	0	11 (31.4)	-
Hospital stay following surgery (days)	19 (15–37)	15 (11–19)	22 (16–50)	0.006

Data are shown as median with interquartile range, except for *: median (range: min–max)

^a^*DG*-Distal gastrectomy

^b^*TG/PG*-Total/proximal gastrectomy

^c^*TG/PG* + *S-*TG/PG plus splenectomy

^d^*RG*-Robotic gastrectomy

^e^*LG*-Laparoscopic gastrectomy

### Risk factors for postoperative complications

To explore the risk factors for postoperative complications, a univariate analysis was performed using the following variables: age ≥ 75 years, male sex, body mass index ≥ 25, scirrhous, bulky node, laparoscopic surgery, TG/PG + S, esophagogastric junction cancer, and conversion surgery (Table 3). The results revealed that LG (*P* = 0.014) or TG/PG + S (*P* < 0.001) were significant risk factors for postoperative complications. With the variables of laparoscopic surgery, and TG/PG + S, which were considered to be related to the risk factor of postoperative complications, multivariate analysis was performed. It was revealed that only TG/PG + S was an independent risk factor [odds ratio, 8.574; 95% confidence interval (CI), 2.584–28.443; *P* < 0.001].

**Table 3:** Univariate/multivariate analyses for risk factors of postoperative complications (grade ≥ 3a)

Variable	Univariate analysis		Multivariate analysis	
	*P*-value	Odds ratio [95% CI^a^]	*P*-value	Odds ratio [95% CI^a^]
Age ≥ 75 years	0.841	0.804 [0.096–6.747]		
Male	0.521	1.468 [0.454–4.740]		
BMI^b^ ≥ 25 kg/m^2^	0.62	1.354 [0.408–4.490]		
Esophagogastric junction cancer	0.588	1.450 [0.378–5.567]		
Scirrhous	0.464	0.459 [0.057–3.699]		
Bulky node	0.635	1.308 [0.431–3.963]		
Laparoscopic surgery	0.014	6.667 [1.474–30.159]	0.052	4.683 [0.984–22.291]
TG^c^/PG^d^ + Splenectomy	< 0.001	10.500 [3.230–34.136]	< 0.001	8.574 [2.584–28.443]
Conversion surgery	0.118	0.298 [0.065–1.358]		

^a^*CI* Confidence interval

^b^*BMI*-Body mass index

^c^*TG*-Total gastrectomy

^d^*PG*-Proximal gastrectomy

### Long-term outcomes

**Fig. 1:**
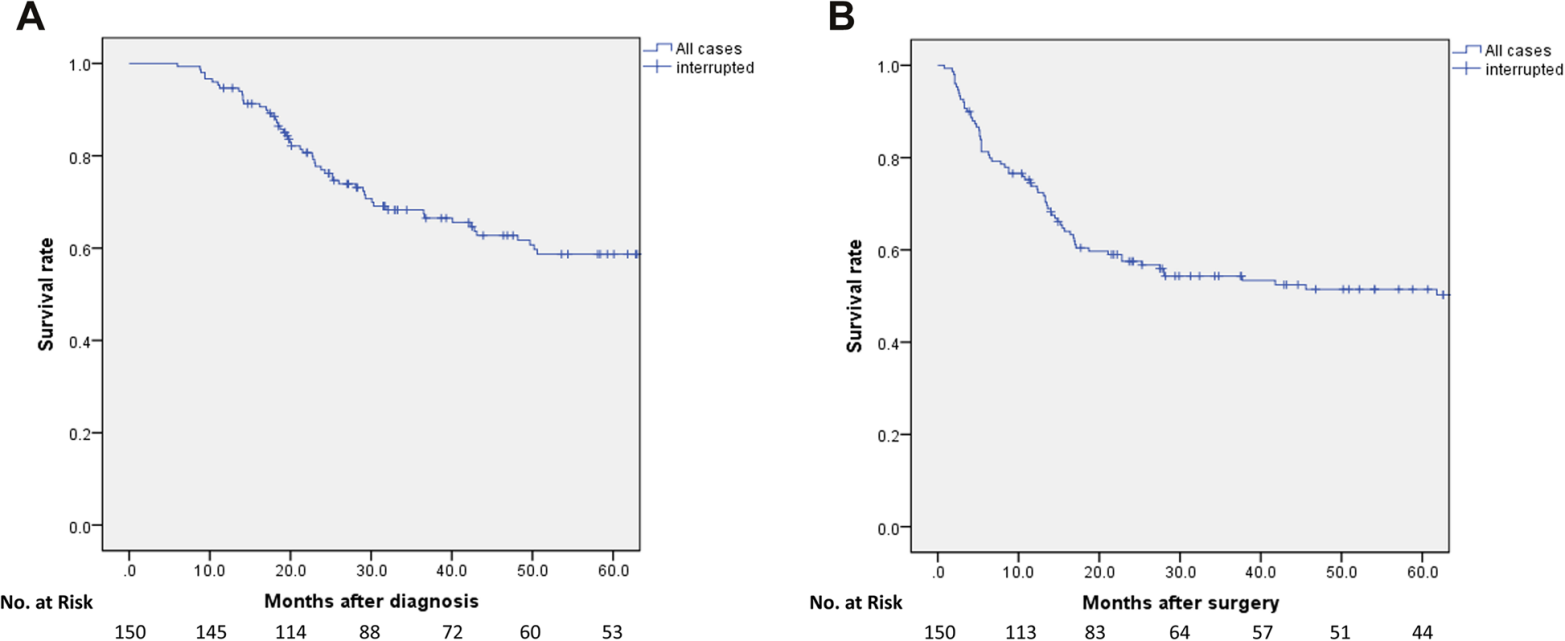
Kaplan–Meier survival curves. Overall survival curves (**a**) and relapse-free survival curves (**b**) for the 150 patients

The long-term outcomes are summarized in Table 4 and electronic supplementary material (Suppl. Figure 2a-f, Suppl. Table 3). The median observation period was 36.7 (IQR: 20.1–81.4) months. Overall, 67 patients (44.7%) developed tumor recurrence during the observation period, including 21 patients (14.0%) with hematogenous metastasis, 15 patients (10.0%) with lymphatic metastasis, and 44 patients (29.3%) with peritoneal metastasis. Tumor recurrence was frequently observed in patients with local complications (75% vs. 41%, *P* = 0.015). The 3- and 5-year OS (RFS) rates of all patients were 68.2% (54.3%) and 58.7% (51.5%), respectively (Fig. 1a, b).

**Table 4:** Long-term outcomes of 150 patients

		Total *n* = 150	Local complication ( +) *n* = 16	Local complication ( −) *n* = 134	*P*-value
Tumor recurrence	n (%)	67 (44.7)	12 (75.0)	55 (41.0)	0.015
Hematogenous metastasis	n (%)	21 (14.0)	5 (31.3)	16 (11.9)	0.051
	liver/bone/lung/brain/ovary/muscle/skin	17/2/2/2/1/1/1	4/0/0/1/0/1/0	13/2/2/1/1/0/1	-
Lymphatic metastasis	n (%)	15 (10.0)	3 (18.8)	12 (9.0)	0.203
	regional/extra-regional	2*/14*	0/3	2*/11*	-
Peritoneal metastasis	n (%)	44 (29.3)	7 (43.8)	37 (27.6)	0.244
Outcomes within 5 years	alive/dead/unknown	78/59/13	4/11/1	74/48/12	0.038

## Discussion

The results of this investigation demonstrated the feasibility of RG for patients with highly advanced gastric cancer who underwent preoperative chemotherapy. In addition, in this cohort, a reduction in the rate of postoperative local complications and tumor stage were identified as prognostic factors. There were three major findings in this study.

First, minimally invasive surgery following preoperative chemotherapy was safe and feasible in this cohort. Recently, the use of minimally invasive surgery has been expanded widely, based on the evidence yielded by clinical trials [15, 39]. In particular, it has been shown that RG is associated with a reduction in the rate of postoperative complications; thus it has attracted considerable attention [17, 21]. In this study, although the cohort included cases of conversion surgery that are typically associated with a higher risk of operative complications, the morbidity rate of minimally invasive gastrectomy was feasible (12.0%), compared to the clinical trials that adopted open surgery in which the morbidity rate (CD grade ≥ 3a) was 10.2%–30.6% [9, 10, 12, 40], and the mortality rate was 0%–2% [40, 41]. In this study, the rate of morbidity of RG in this cohort (3.2%) was acceptable and comparable to that reported in a multicenter study of patients with cStage 1–2 who underwent RG (2.5%) [17]. Specifically, there were no significant differences for non-splenectomy cases in short-term postoperative outcomes between RG and LG, regardless of the DG resection type or TG/PG. This indicated that the surgeon’s qualification regarding gastrectomy following preoperative chemotherapy in our institute was appropriate based on previously reported necessary surgical volume and surgeon’s qualifications for safer minimally invasive surgery [42, 43]. Therefore, we concluded that our surgical concepts, such as outermost layer-oriented nodal dissection and the technique of splenectomy, were acceptable in technically demanding cases receiving preoperative chemotherapy. Moreover, the use of DVSS could further reduce the rate of postoperative complications and shorten the duration of hospital stay after surgery, especially for splenectomy cases, which could outweigh the disadvantage of the high cost.

Second, we revealed that the laparoscopic approach or TG/PG + S was a significant risk factor for postoperative complications. Previous reports revealed that splenectomy or LG was a risk factor for pancreatic fistula [44, 45]. In our series, splenectomy was less common in the RG group versus the LG group due to differences in historical background. However, consistent results were observed in the sub-analysis of patients who underwent splenectomy. These results highlighted the excellent operability of DVSS with regard to multi-articulated joint angle and near-infrared fluorescent imaging, which enables us to confirm lymphatic flow or blood supply. It may further attenuate the risk of pancreatic fistula occurrence such that we could easily transform the procedure in the same manner in which we preserve the caudal artery after confirming insufficient blood perfusion to the pancreatic tail. This enables us to achieve more precise and safer dissection even in patients with highly advanced cancer after chemotherapy [35]. Although the use of prophylactic splenectomy has been decreasing according to the results of the JCOG 0110 study [45], it remains necessary for cases with invasion of the greater curvature, a positive hilum node, or direct invasion [46, 47]. Therefore, robotic surgery may be useful in such technically demanding cases, particularly in therapeutic splenic hilum nodal dissection.

Third, the occurrence of a local complication was associated with poor prognosis in terms of disease recurrence; this finding was consistent with the results of other studies [48–50]. Remarkably, in this cohort, the rate of hematological metastasis in patients who developed local complications was higher than that recorded in patients who did not. A potential explanation for this observation is that tumor recurrence might be affected by surgical invasiveness, local complications, and tumor characteristics. RG may reduce the rate of local complications in patients with highly advanced gastric cancer. In turn, this effect may enhance recovery after surgery and improve compliance with adjuvant chemotherapy. Based on our results, it is necessary to focus on surgical invasiveness, which might have an impact on oncological outcomes, as well as explore more powerful regimens. Therefore, prospective studies are required to confirm the efficacy of aggressive perioperative chemotherapy and minimally invasive surgery using a surgical robotic system.

This study had several limitations. First, this was a retrospective investigation conducted at a single institution. Second, the sample size was small, and the interruption of observation may have affected the reported OS rates. Third, the accumulation of clinical expertise in surgical techniques might influence operative outcomes regarding postoperative complications. Fourth, the cutoff for tumor size was set at ≥ 5 cm for preoperative chemotherapy in this cohort; however, the prognosis of patients with a tumor diameter of ≥ 8 cm was found to be significantly worse. Thus, the cutoff for the diameter of large tumors needs to be reviewed, considering this subgroup analysis. Fifth, we could not sufficiently evaluate long-term outcomes due to the short observation period and variability in tumor stage and chemotherapy regimens. In addition, we evaluated the neoadjuvant group in combination with the conversion group, making it difficult to interpret the data; however, the prognosis of patients who obtained a favorable response (grade ≥ 2) in this cohort was similar to that of the neoadjuvant group. We believe that stronger regimens can attenuate the differences between neoadjuvant and induction chemotherapy groups. A prospective, larger-scale study is required to confirm the advantage of minimally invasive surgery and the efficacy of preoperative chemotherapy for highly advanced gastric cancer.

In conclusion, minimally invasive gastrectomy following preoperative chemotherapy was feasible and safe for patients with highly advanced gastric cancer. RG may improve surgical safety, particularly in the case of TG/PG + S. In combination with more effective regimens of preoperative chemotherapy, RG may play an important role in multidisciplinary treatment in the future.

### Supplementary Information

**Additional file 1:** **Supplementary Figure 1.** Consort diagram. **Supplementary Figure 2.** Kaplan–Meier survival curves for overall survival (subgroups). **Supplementary Table 1.** Adverse events during preoperative chemotherapy. **Supplementary Table 2.** Short-term outcomes and risk factors for postoperative complications following gastrectomy. **Supplementary Table 3.** Prognostic risk factors (All cases).

## Data Availability

The datasets generated and analyzed during the current study are available from the corresponding author on reasonable request.
