# Supplementary material for: Safety and feasibility of minimally invasive gastrectomy following preoperative chemotherapy for highly advanced gastric cancer
Source: BMC Gastroenterol. 2024 Feb 15;24:74. doi: 10.1186/s12876-024-03155-5 (PMC10870591; doi:10.1186/s12876-024-03155-5)
Supplement: Supplementary file 1 — Additional file 1: Supplementary Figure 1. Consort diagram. Supplementary Figure 2. Kaplan–Meier survival curves for overall survival (subgroups). Supplementary Table 1. Adverse events during preoperative chemotherapy. Supplementary Table 2. Short-term outcomes and risk factors for postoperative complications following gastrectomy. Supplementary Table 3. Prognostic risk factors (All cases). [file 12876_2024_3155_MOESM1_ESM.docx]

**Electronic Supplementary Material**

**Article title:** Safety and feasibility of minimally invasive gastrectomy following preoperative chemotherapy for highly advanced gastric cancer

**Journal name:** BMC Gastroenterology

**Author names:** Tsuyoshi Tanaka, MD, PhD; Koichi Suda, MD, PhD; Susumu Shibasaki, MD, PhD; Akiko Serizawa, MD, PhD; Shingo Akimoto, PhD; Masaya Nakauchi, MD, PhD; Hiroshi Matsuoka^1^, MD, PhD, Kazuki Inaba, MD, PhD; and Ichiro Uyama, MD, PhD

**Corresponding author:** Koichi Suda, MD, PhD

Department of Surgery, Fujita Health University

E-mail: ko-suda@nifty.com

**Supplementary Figure 1** Consort diagram


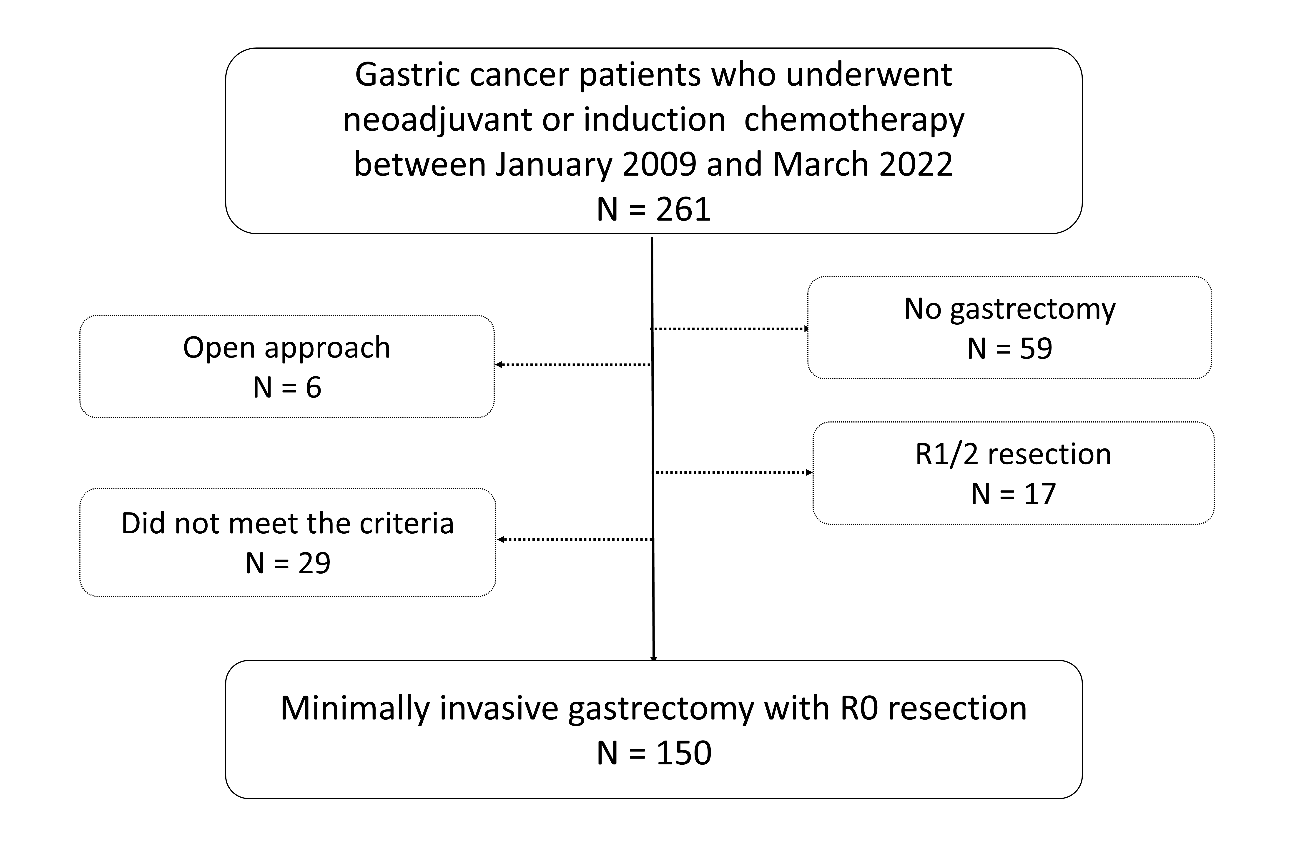


Between January 2009 and March 2022, 261 consecutive patients with gastric cancer, including cStage IV cancer, underwent neoadjuvant or induction chemotherapy. Among these, 150 patients who met the criteria of this study were analyzed.

**Supplementary Figure 2** Kaplan–Meier survival curves for overall survival (subgroups)

**
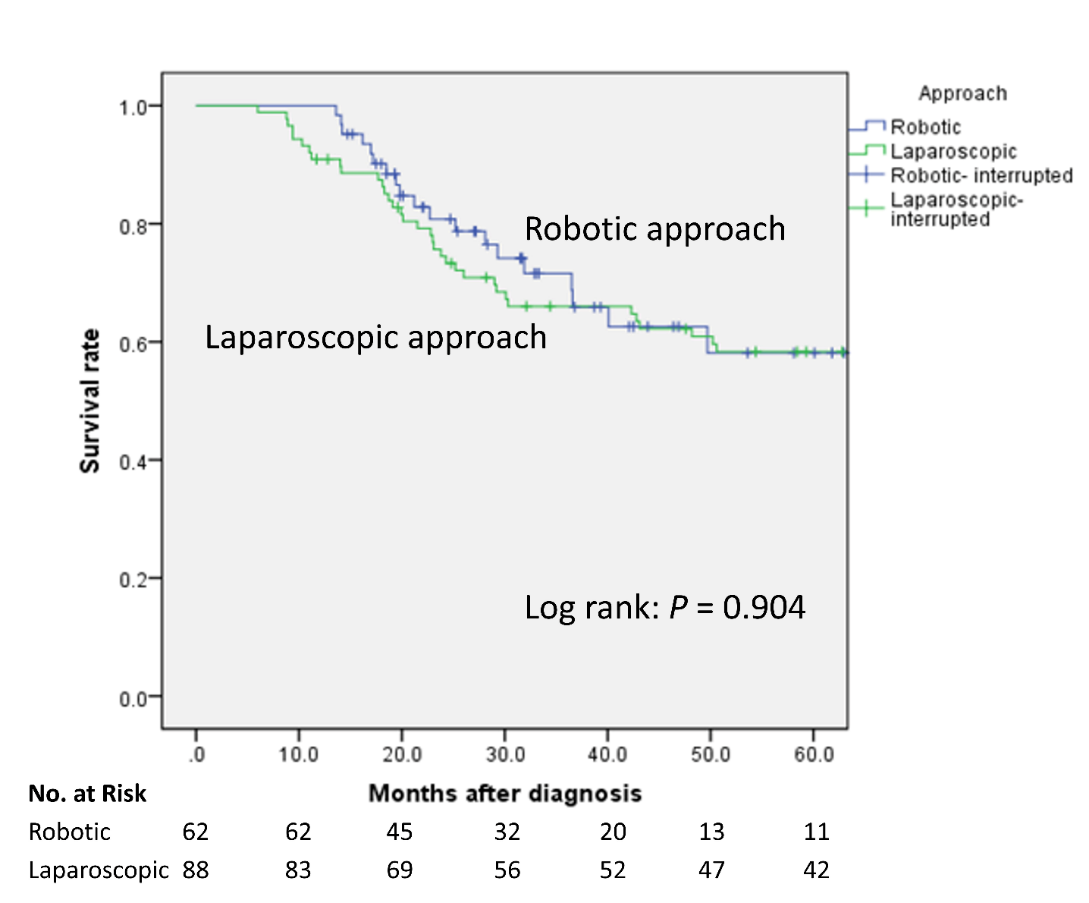
**

**a)**

**
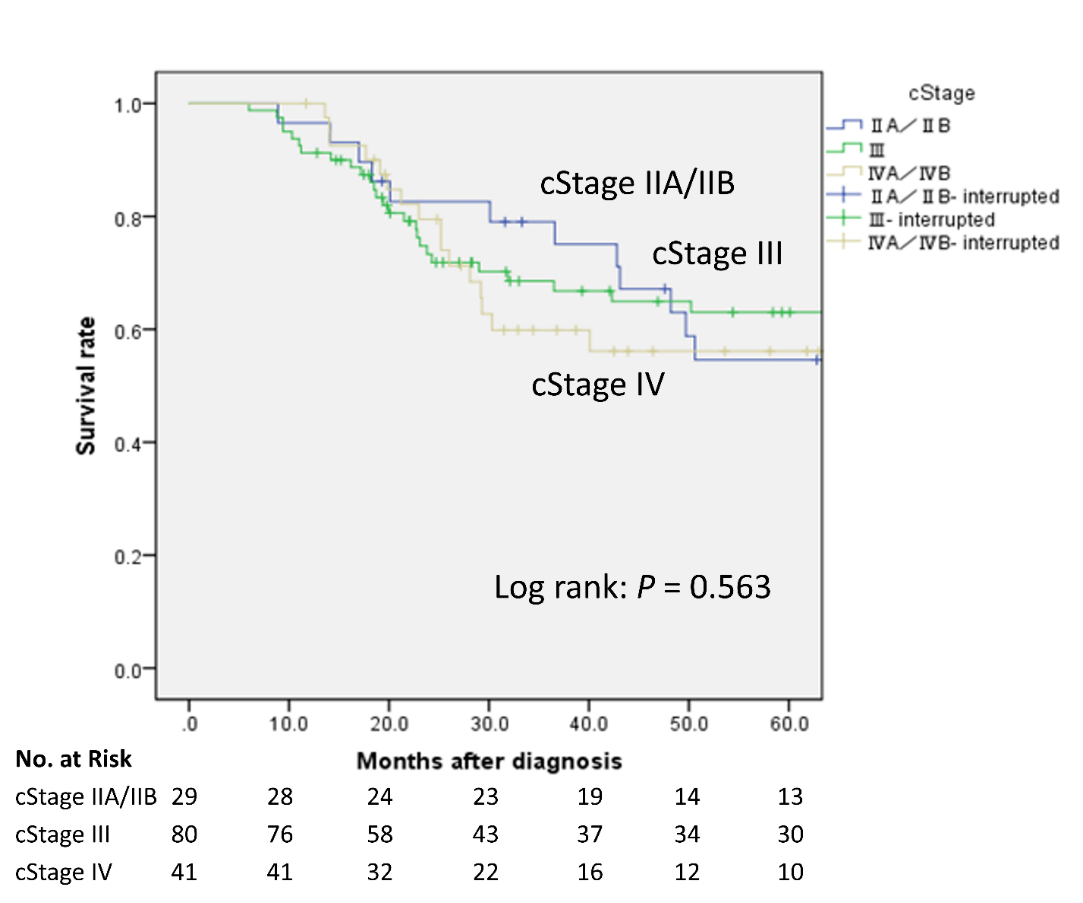
**

**b)**


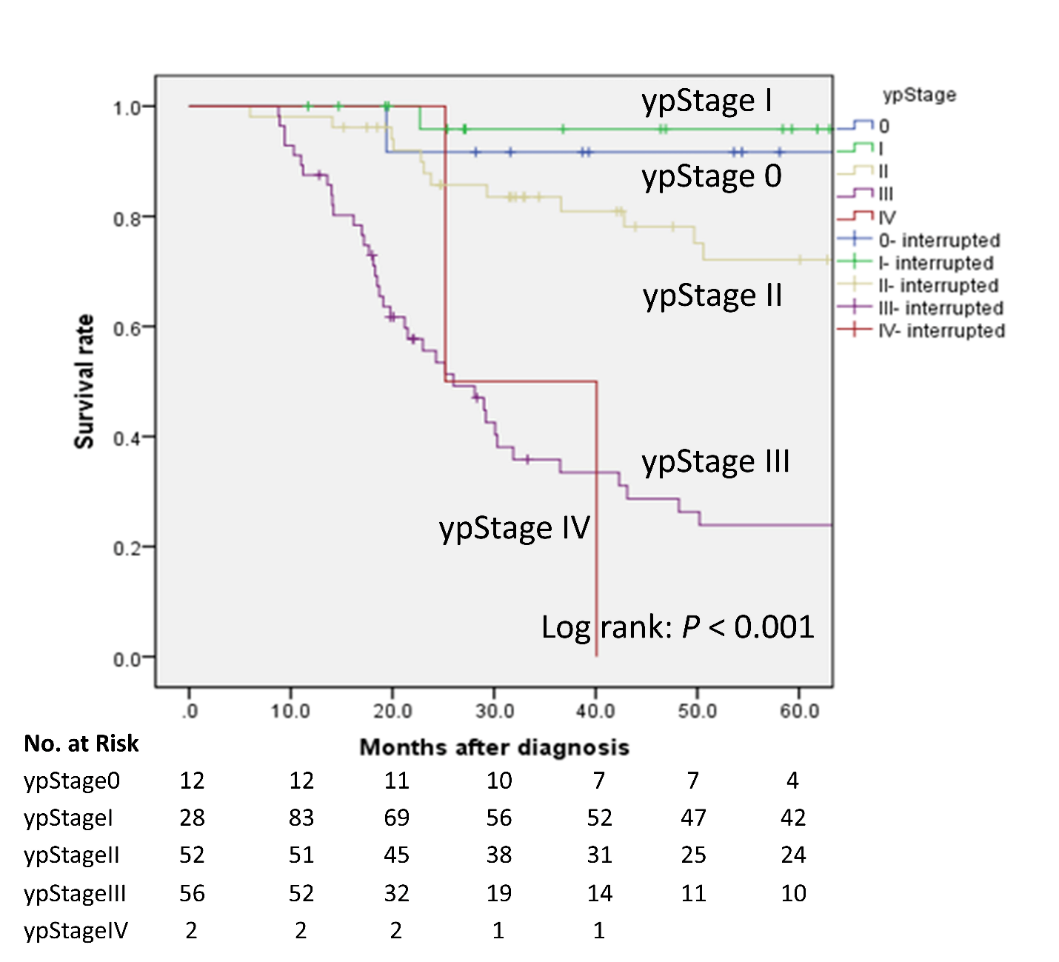


**c)**


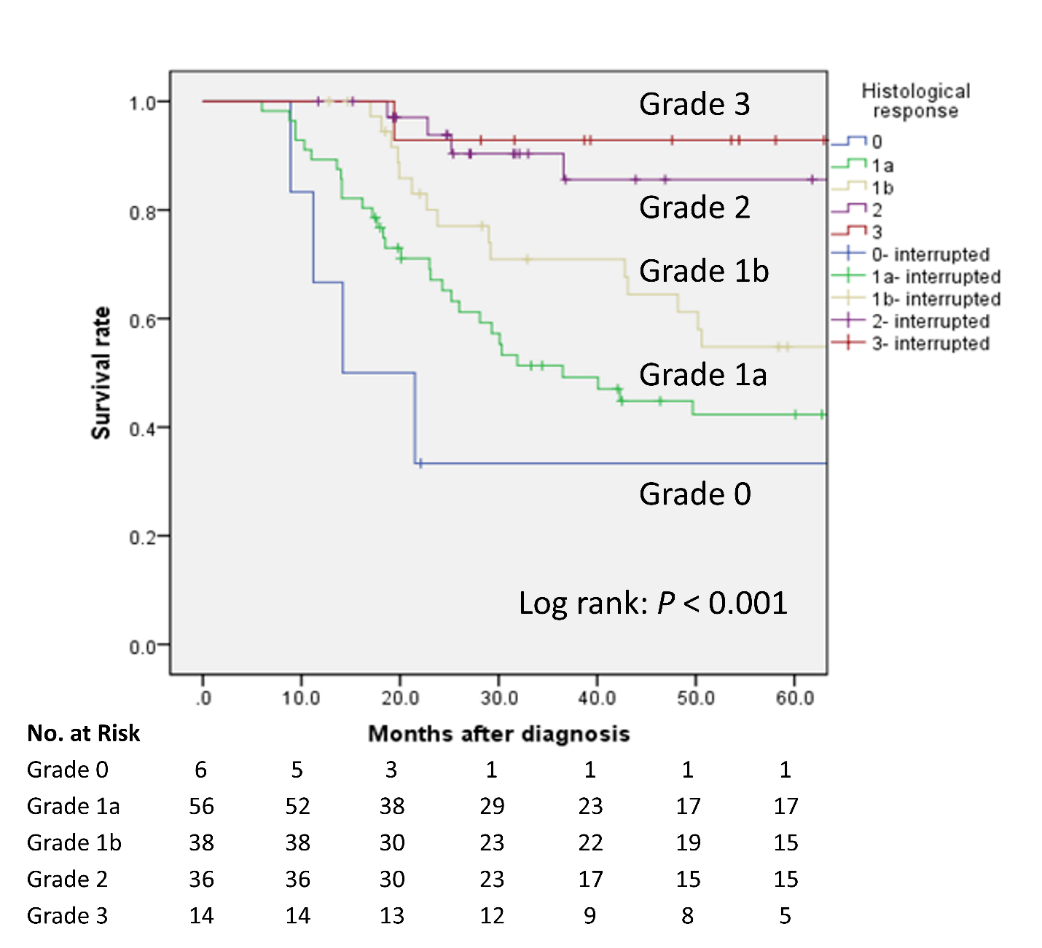


**d)**


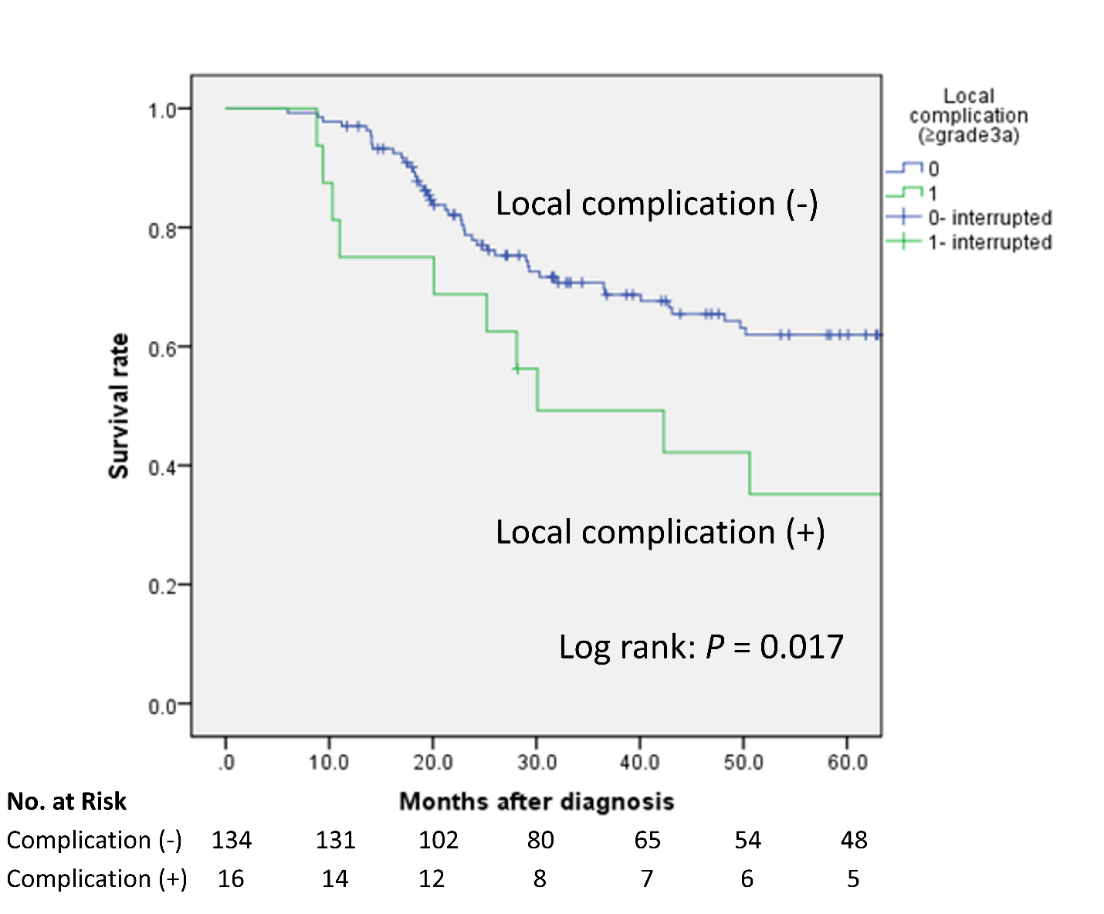


**e)**

**
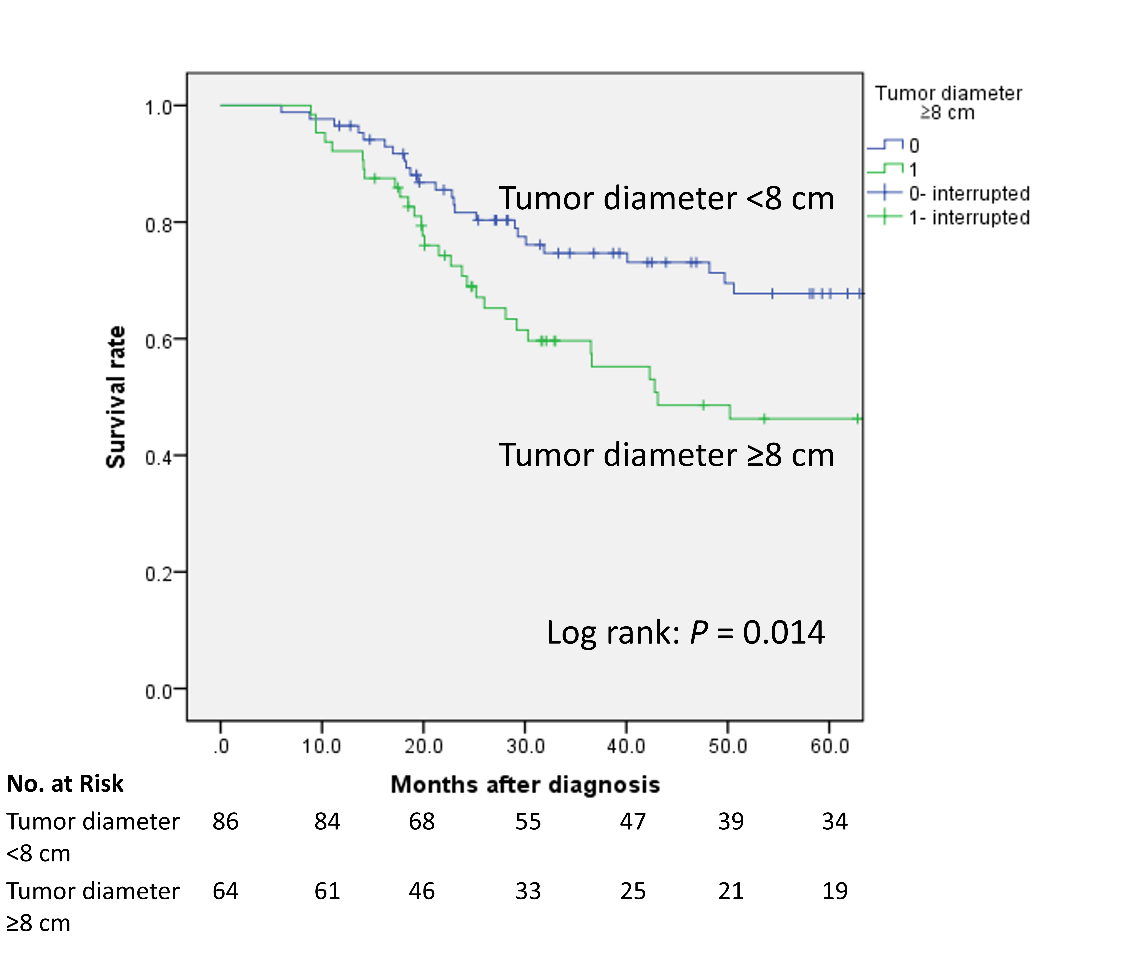
**

**f)**

a. Overall survival (OS) stratified by approach (robotic/laparoscopic). b. OS stratified by cStage. c. OS stratified by ypStage. d. OS stratified by histological response. e. OS stratified by the presence of local complications. f. OS stratified by tumor diameter.

Kaplan–Meier survival curves for OS were found to be well stratified by pathological stage (ypStage) (*P* < 0.001), histological response (*P* < 0.001), local complications (grade ≥3a) (*P* = 0.019), and tumor diameter (*P* = 0.014). However, they were not well stratified by operative approach (*P* = 0.904) and cStage (*P* = 0.563) (Suppl. Fig. 2a–2f).

**Supplementary Table 1** Adverse events during preoperative chemotherapy

|  | Grade | | | | % Grade ≥3 |
| --- | --- | --- | --- | --- | --- |
|  | 1 | 2 | 3 | 4 |  |
| **Hematological toxicity** |  |  |  |  |  |
| Anemia | 69 | 61 | 8 | 0 | 5.3 |
| Leucopenia | 38 | 28 | 14 | 1 | 10.0 |
| Neutropenia | 25 | 30 | 23 | 9 | 21.3 |
| Febrile neutropenia | 0 | 0 | 3 | 0 | 2.0 |
| Thrombocytopenia | 86 | 16 | 2 | 2 | 2.7 |
| Elevated level of aspartate aminotransaminase | 57 | 4 | 0 | 0 | 0.0 |
| Elevated level of alanine aminotransferase | 62 | 4 | 2 | 0 | 1.3 |
| Elevated level of blood bilirubin | 21 | 4 | 0 | 0 | 0.0 |
| Elevated level of creatinine | 49 | 14 | 0 | 0 | 0.0 |
| Hyperkalemia | 34 | 0 | 0 | 0 | 0.0 |
| Hyponatremia | 83 | 7 | 2 | 1 | 2.0 |
| Hypokalemia | 34 | 2 | 5 | 1 | 4.0 |
| **Nonhematological toxicity** |  |  |  |  | 0.0 |
| Nausea | 56 | 24 | 10 | 0 | 6.7 |
| Vomiting | 21 | 7 | 1 | 0 | 0.7 |
| Loss of appetite | 61 | 26 | 12 | 0 | 8.0 |
| Fatigue | 48 | 16 | 2 | 0 | 1.3 |
| Weight loss | 37 | 15 | 0 | 0 | 0.0 |
| Diarrhea | 27 | 13 | 16 | 0 | 10.7 |
| Constipation | 22 | 3 | 0 | 0 | 0.0 |
| Colitis | 0 | 2 | 0 | 0 | 0.0 |
| Oral mucositis/pharyngeal mucositis | 21 | 9 | 0 | 0 | 0.0 |
| Dysgeusia | 45 | 1 | 0 | 0 | 0.0 |
| Hiccups | 16 | 8 | 0 | 0 | 0.0 |
| Abdominal pain | 16 | 8 | 1 | 0 | 0.7 |
| Peripheral neuropathy | 34 | 6 | 0 | 0 | 0.0 |
| Skin disorders | 27 | 16 | 1 | 0 | 0.7 |
| Fever | 22 | 1 | 1 | 0 | 0.7 |
| Alopecia | 7 | 4 | 0 | 0 | 0.0 |
| Edema | 14 | 0 | 0 | 0 | 0.0 |
| Headache | 8 | 1 | 0 | 0 | 0.0 |
| Dizziness | 7 | 1 | 0 | 0 | 0.0 |
| Syncope | 0 | 0 | 4 | 0 | 2.7 |
| Epistaxis | 6 | 0 | 0 | 0 | 0.0 |
| Allergic reaction | 1 | 1 | 0 | 0 | 0.0 |
| Hypertension | 1 | 1 | 0 | 0 | 0.0 |
| Arrythmia/Palpitations | 1 | 1 | 0 | 0 | 0.0 |
| Arthralgia | 1 | 1 | 0 | 0 | 0.0 |
| Phlebitis | 0 | 1 | 0 | 0 | 0.0 |
| Thromboembolic events | 0 | 0 | 1 | 0 | 0.7 |
| Urinary tract infection | 0 | 0 | 1 | 0 | 0.7 |

**Supplementary Table 2:** Short-term outcomes and risk factors for postoperative complications following gastrectomy

|  | Total (n = 150) |
| --- | --- |
| Number of surgeons | 18 |
| Number of cases operated upon by each surgeon | 3.5 (1–39)* |
| Operative time (min) | 475 (360–592) |
| Blood loss (g) | 72 (26–170) |
| Number of dissected nodes | 41 (32–51) |
| In-hospital mortality, n (%) | 0 |
| Morbidity (grade ≥3a), n (%) | 18 (12.0) |
| Systemic complications, n (%) | 5 (3.3) |
| *Pneumonia* | *4* |
| *Pulmonary thromboembolism* | *1* |
| *Sepsis* | *1* |
| *Renal failure* | *1* |
| Local complications, n (%) | 16 (10.7) |
| *Pancreatic fistula* | *11* |
| *Ileus* | *3* |
| *Anastomotic leakage* | *2* |
| *Abdominal abscess* | *2* |
| *Choledochiarctia* | *1* |
| *Diaphragmatic hernia* | *1* |
| Local infectious complications, n (%) | 12 (8.0) |
| Hospital stay following surgery (days) | 14 (11–18) |

Data are shown as median with interquartile range, except for *: median (range: min–max).

**Supplementary Table 3** Prognostic risk factors (All cases)

|  | Univariate analysis | | | Multivariate analysis | | |
| --- | --- | --- | --- | --- | --- | --- |
| Variables | *P*-value | Hazard ratio | 95% CI^[[1]](#footnote-2)^ | *P*-value | Hazard ratio | 95% CI |
| Overall survival |  |  |  |  |  |  |
| Age ≥ 75 years | 0.662 | 1.227 | 0.491–3.070 |  |  |  |
| ASA-PS^[[2]](#footnote-3)^ ≥ 3 | 0.998 | 1.001 | 0.362–2.771 |  |  |  |
| Male sex | 0.663 | 0.884 | 0.507–1.540 |  |  |  |
| BMI < 18.5 kg/m^2^ | 0.945 | 0.975 | 0.479–1.985 |  |  |  |
| Esophagogastric junction cancer | 0.631 | 1.201 | 0.569–2.534 |  |  |  |
| Tumor diameter ≥ 8 cm | 0.016 | 1.884 | 1.127–3.149 | 0.210 | 1.428 | 0.818–2.492 |
| Scirrhous | <0.001 | 3.258 | 1.709–6.209 | 0.002 | 2.835 | 1.450–5.542 |
| Bulky node metastasis | 0.803 | 1.08 | 0.592–1.969 |  |  |  |
| cStage IV | 0.375 | 1.287 | 0.737–2.249 | 0.197 | 1.477 | 0.816–2.672 |
| Laparoscopic approach | 0.904 | 0.967 | 0.559–1.672 |  |  |  |
| Histological response grade < 2 | <0.001 | 6.588 | 2.635–16.475 | <0.001 | 5.715 | 2.271–14.382 |
| Morbidity grade ≥ 3a | 0.119 | 1.687 | 0.875–3.253 |  |  |  |
| Local complication grade ≥ 3a | 0.020 | 2.173 | 1.128–4.188 | 0.013 | 2.360 | 1.201–4.637 |

The univariate analysis demonstrated that tumor diameter ≥ 8 cm (*P* = 0.016), scirrhous (*P* < 0.001), histological response grade < 2 (*P* < 0.001), and local complication grade ≥ 3a (*P* = 0.023) were significantly associated with overall survival (OS). Multivariate analysis was performed for the variables that were expected to be related to prognosis, including tumor diameter ≥ 8 cm, scirrhous, cStage IV, histological response grade < 2, and local complication grade ≥ 3a. The findings revealed that scirrhous (hazard ratio [HR], 2.835; 95% confidence interval [CI], 1.450–5.542; *P* = 0.002], histological response grade < 2 (HR, 5.715; 95% CI, 2.271–14.382; *P* < 0.001], and local complications (grade ≥ 3a) [HR, 2.36; 95% CI, 1.201–4.637; *P* = 0.013] were the independent prognostic factors for OS.

1. *CI-*Confidence interval [↑](#footnote-ref-2)
2. *ASA-PS*-American Society of Anesthesiologists-Physical Status [↑](#footnote-ref-3)
